# The relationship between core temperature and perioperative shivering during caesarean section under intrathecal anesthesia with bupivacaine and ropivacaine: a randomized controlled study

**DOI:** 10.1007/s00540-021-02995-9

**Published:** 2021-09-02

**Authors:** Guangju Feng, Yu Wang, Jiehua Feng, Xiaomin Luo, Chaoyang Li, Shanglong Yao

**Affiliations:** 1 Department of Anesthesiology, Huazhong University of Science and Technology Union Shenzhen Hospital, No. 89, Tao yuan road, Nanshan, Shenzhen, Guangdong China; 2 Department of Anesthesiology, Union Hospital, Tongji Medical College, Huazhong University of Science and Technology, No. 1277, Jiefang Avenue, Wuhan, 430022 China

**Keywords:** Bupivacaine, Ropivacaine, Shivering, Caesarean section, Spinal anesthesia

## Abstract

**Purpose:**

To assess the incidence rate of perioperative shivering for cesarean section and explore the associations between the occurrence of shivering and hypothermia, core temperature change, local anesthetic.

**Methods:**

This is a prospective, randomized, controlled, double-blinded study of 100 patients consenting for caesarean section under intrathecal anesthesia. Parturients with ASA I or II accepted elective caesarean section with combined spinal-epidural anesthesia (SA). 2–2.5 ml of 0.5% bupivacaine or 0.5% ropivacaine was intrathecally injected in group B and group R, respectively.

**Results:**

The intraoperative shivering incidence in group B was significantly higher than that in group R (66.7 vs. 20.5%, *P*value < 0.001), and shivering intensity in group B was significantly greater than group R (score: 1.4 vs. 0.3, *P*value < 0.001). The core temperature in both groups gradually decreased with the time after SA. Hypothermia (core temperature < 36.0 ℃) 5–30 min after SA was not associated with shivering. However, changes of temperature at 25 and 30 min after SA, and bupivacaine were statistically associated with shivering, with the odds of 10.77 (95% CI: 1.36–85.21, *P* value = 0.02), 8.88 (95% CI: 1.29–60.97, *P* value = 0.03), and 7.78 (95% CI: 2.94–20.59, *P* value < 0.01), respectively.

**Conclusions:**

In our study, for cesarean section, the occurrence of shivering was associated with the local anesthetics and the change of core temperature after SA, while not the hypothermia.

## Introduction

As a prevalent and well-accepted technique for cesarean section, spinal-epidural anesthesia (SA) has certain advantages such as rapid onset, high success rate, less maternal and fetal side effects with minimal maternal discomfort over general anesthesia [1]. Perioperative shivering during SA is a common problem, occurring up to 40–80% incidence rate for cesarean section [2]. Shivering is uncomfortable and can interfere with patient monitoring. It could change the patient’s physiology by increasing oxygen consumption, increase the depth of pain, affect the parturient's delivery experience [3, 4], as well as many postoperative complications such as surgical site infection, myocardial ischemia, and peri-operative coagulopathy [5].

Preoperative warming strategies to reduce shivering include adjusting temperature or density of intrathecal anesthetics, administering intrathecal opioids, warming intravenous fluid, as well as warming devices including lower body forced-air warmer and resistive warming mattresses [6–9]. Nevertheless, the problem and importance of shivering appear to be underrated in both the literature and in everyday clinical practice.

Shivering is thought to be related to intraoperative hypothermia [10], but its risk factors and exact mechanism are still unclear [11]. One recent study explored the risk factors for shivering during caesarean section under spinal anesthesia [12]. They failed to prove a strong correlation between some important variables and shivering, including hypothermia, temperature difference during surgery, type of local anesthetics, and so on. Whether hypothermia or core temperature variability causes shivering is still worthy of further study. On the other hand, the most important cause of heat redistribution from the core to the periphery due to vasodilation is the induction of general or neuraxial anesthesia [13]. According to the efficacy of local anesthetics, bupivacaine and ropivacaine are most widely used in spinal anesthesia [14, 15]. All of these point together towards a need for a comprehensive understanding of the relationship between hypothermia, core temperature change, local anesthetics, and shivering. We, therefore, conducted a prospective, double-blind, and randomized study on parturients accepted caesarean section under spinal anesthesia with bupivacaine and ropivacaine. Our study was aimed to explore the associations between the occurrence of shivering and hypothermia, core temperature change, local anesthetic.

## Materials and methods

### Study design

After approval from the ethics committee and obtaining the written informed consent, 100 parturients were enrolled in our study during the period from May 2017 to March 2018. The inclusion criteria were at the age of 25–40 years; weight 50–80 kg; with American Society of Anaesthesiologist (ASA) physical status I or II; scheduled for elective caesarean section under SA. The parturients who refused to participate, having fever, pregnancy-induced hypertension, obesity (body mass index > 35 kg•m^−2^), and failure of SA requiring conversion to general anesthesia, or requiring blood transfusion were excluded from the study.

### Randomization and blinding

After obtaining informed consent, subjects were allocated to two groups (group B and group R) by computer-generated random assignment placed in sequentially numbered sealed opaque envelopes. Randomization is based on the patient's enrollment order and the random code generated in SAS statistical software (version 9.4, SAS Institute, Cary, NC, USA) using simple randomization. The patients, anesthesiologists, and outcome assessors were blinded to the randomization assignment.

### Anesthesia protocol and monitoring

The temperature of the operating room was maintained at 23–25 °C throughout the study. There were no pre-operation medications, and food access was forbidden for 6 h before surgery. All the parturients were placed under standard monitoring with BeneView T5 (MINDRAY, CHINA), received preload warmed colloid (Hydroxyethyl starch 130/0.4 10 mL•kg^−1^), and crystalloid infusion (lactated Ringers solution 10 mL•kg^−1^) for the prevention of hypotension. A fluid warmer (Barkey S-line, Germany) was used for perioperative fluid therapy. A thermometer was inset 7–10 cm to the rectum as the core temperature monitor before the anesthesia.

The patient was in the lateral decubitus position. SA was performed with the patient at L2–L3 or L3–L4 intervertebral space with a 25G Quincke’s spinal needle, using the needle-in-needle technique. Isobaric local anesthetic with normal temperature was used for SA in each group. 2–2.5 mL of 0.5% bupivacaine (determined at the discretion of the attending anesthesiologists) was intrathecally injected with 1 mL per 5–8 s in group B, and 2–2.5 mL of 0.5% ropivacaine was intrathecally injected with the same speed in group R. Then the epidural catheter was inset 3–5 cm to epidural space at the same intervertebral space for post-operative analgesia.

### Outcome assessment

The anesthesiologists who assessed shivering were blinded to the administered drugs. The efficacy of anesthesia was evaluated using the level of maximum sensory block (noted as ‘Tn’ where ‘T’ represents the thoracic intervertebral space and ‘n’ represents the level of the dermatome sensory block) was observed and noted with the pinprick method [13]. Intraoperative shivering incidence rate and degree were graded using a scale validated by Crossley and Mahajan (score 0 = no shivering; score 1 = no visible muscle activity, but one or more of piloerection, peripheral vasoconstriction or peripheral cyanosis (other causes exclude); score 2 = muscular activity in only one muscle group; score 3 = moderate muscular activity in more than one muscle group, but not generalized shaking; score 4 = violent muscular activity that involves entire body) [16]. The intraoperative rectum temperature, heart rate, mean arterial pressure (MAP) were recorded before the beginning of anesthesia (T0) and 5 min (T1), 10 min (T2), 15 min (T3), 20 min (T4), 25 min (T5), 30 min (T6) after anesthesia. Hypothermia was defined as core temperature < 36.0 ℃. Hypotension was defined as a decrease in systolic blood pressure (SBP) to < 90 mmHg or symptomatic decrease (e.g., light-headedness) from baseline and treated with intravenous ephedrine 10 mg at the discretion of the anesthesiologist.

### Sample size

To detect a difference of 30% or greater between groups for shivering incidence, assuming that the incidence of 0.5 in group B and 0.2 in group R, 48 subjects in each group would be needed to reach a power of 95% at *α* = 0.05. The target sample size was 50 patients in each group. We conducted sample size estimation using PASS software (version 15.0, NCSS, Kaysville, USA).

### Statistical analysis

Continuous data were expressed as mean ± standard deviation (SD). Intra-group comparisons were analyzed by using the Turkey t-test or Kruskal–Wallis Test, where appropriate. Categorical variables were described as the number of patients and percentage. They were compared using the chi-square or Fisher exact test in the presence of expected cell frequencies less than 5. We further explored the associations between the occurrence of shivering and hypothermia (core temperature < 36.0 ℃), core temperature change, local anesthetic using the logistic regression. The receiver operating characteristic curve (ROC) curve was also drawn, and the sensitivity, specificity, critical value, and area under the curve (AUC) were calculated. *P* value < 0.05 was considered statistically significant. All statistical analyses were performed using IBM SPSS statistical software (version 22.0, IBM, New York, USA).

## Results

One hundred women met inclusion criteria and were consented to participation. Fourteen patients were removed from the study due to SA failure resulting in need for epidural anesthesia (*n* = 5), pregnancy-induced hypertension (*n* = 3), missing data collection (*n* = 2), and hemorrhage over 800 ml with the need for blood transfusion (*n* = 4). Eighty-six women (group B for 42 and group P for 44) completed the study protocol. The demographic characteristics (age, weight, height, body mass index) showed no statistically significant difference, as well as the volume of drugs (2.3–2.4 mL) and the height of sensory block (T6 to T4) between the two groups (Table 1). 26 patients in group B and 32 patients in group R were above T6. The remaining patients reached T6, and the anesthetic effect of all included patients was satisfactory with no additional anesthetics.

**Table 1:** Demographic data and anesthesia baseline data among 2 groups

Variable	Group B (*n* = 42)	Group R (*n* = 44)	*P* value
Age(yrs)	31.5 ± 3.37	29.7 ± 2.46	0.366
Weight(kg)	68.5 ± 9.43	72.7 ± 6.94	0.056
Height (cm)	161.0 ± 3.18	161.3 ± 3.20	0.719
Body mass index (kg•m^−2^)	26.5 ± 3.68	27.0 ± 3.23	0.059
Dose of drugs	2.3 ± 0.21	2.4 ± 0.12	0.060
Sensory block height (above T6)	26, 61.9%	32, 72.7%	0.359

Continuous data were expressed as mean ± SD, Categorical variables were described as the number of patients and percentage

The MAP values were decreased in both groups at T1 (group B: 77 ± 7.0 vs. 62 ± 5.1 and group R: 76 ± 5.8 vs. 65 ± 3.1, *P* value < 0.01). There was no difference in HR and MAP at other time points in both groups (Table 2).

**Table 2:** The HR and MAP change duration of surgery among 2 groups

	T0	T1	T2	T3	T4	T5	T6
HR							
Group B	78 ± 6.2	75 ± 7.1	71 ± 7.2	74 ± 7.6	79 ± 4.5	82 ± 7.4	81 ± 2.2
Group R	78 ± 6.4	75 ± 7.1	72 ± 7.0	74 ± 7.7	80 ± 4.6	83 ± 7.7	81 ± 2.2
P	0.779	0.716	0.213	0.770	0.718	0.994	0.691
MAP							
Group B	77 ± 7.0	62 ± 5.1**	69 ± 3.0	74 ± 3.4	79 ± 4.0	83 ± 5.4	84 ± 4.4
Group R	76 ± 5.8	65 ± 3.1**	69 ± 2.7	73 ± 2.5	78 ± 3.4	82 ± 4.4	83 ± 3.8
*P*	0.527	< 0.01	0.373	0.073	0.213	0.288	0.357

*P* value was used to indicate the difference between the HR and MAP at T0 and after T0 among group B and R

^**^Significant (*P* < 0.01)

After SA, the intraoperative shivering incidence in group B was significantly higher than that in group R (66.7 vs. 20.5%, *P* value < 0.001), and shivering intensity in group B was significantly greater than group R (score: 1.4 ± 1.4 vs. 0.3 ± 0.6, *P* value < 0.001) (Table 3).

**Table 3:** Comparison of incidence and grade of shivering

Group	Grade of shivering					Shivering incidence	Shivering intensity	*P* value
	0	1	2	3	4			
B	14	13	4	5	6	28/42	1.4 ± 1.4	< 0.001
R	35	6	3	0	0	9/44	0.3 ± 0.6	

*P* values for both incidence and intensity of shivering among Group B and R were < 0.001

**Fig. 1:**
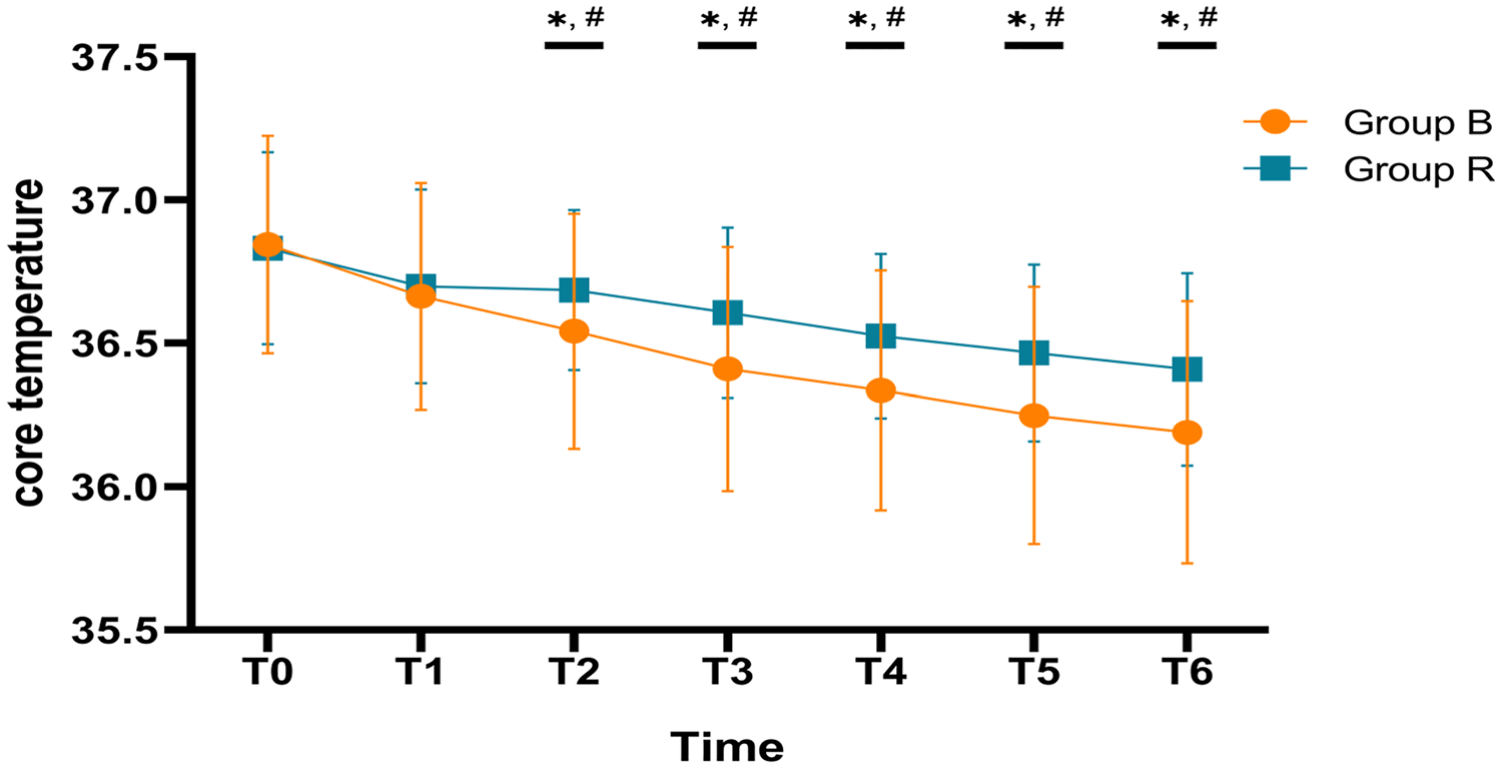
The core temperatures at different time among group B and R. Multiple comparisons between temperatures after anesthesia and temperatures at T0 were conducted using Turkey honest significant difference test; * was used to indicate the significant difference between the tempratures at T0 and after T0 in group B (P-value < 0.05); # was used to indicate the significant difference between the tempratures at T0 and after T0 in group R (*P* value < 0.05)

The core temperature of patients in group B and R was significantly lower after SA than that at T0, whereas core temperature values did not change significantly at T1 (Fig. 1). These results indicated that the core temperatures decrease significantly after 10 min of SA. As Fig. 1 showed, the core temperatures gradually decreased with the time after SA.

The logistic regression results showed the relationship between the core temperature, local anesthetic, and shivering (Table 4). Patients in group B were more prone to shivering, with the odds of 7.78 (95% CI: 2.94–20.59, *P* value < 0.01). Hypothermia (core temperature < 36.0 ℃) 5–30 min after SA was not statistically significantly associated with shivering. However, changes in temperature at T5 and T6 were statistically associated with shivering, with the odds of 10.77 (95% CI: 1.36–85.21, *P* value = 0.02) and 8.88 (95% CI: 1.29–60.97, *P* value = 0.03), respectively.

**Table 4:** The relationship between the core temperature, local anesthetic and the shivering

	Odds	95% CI	*P* value
SA (reference = group R)	7.78	2.94–20.59	0.00*
Hypothermia at T1	1.34	0.18–10.00	0.77
Hypothermia at T2	2.74	0.24–31.46	0.42
Hypothermia at T3	2.97	0.69–12.76	0.14
Hypothermia at T4	0.41	0.08–2.16	0.29
Hypothermia at T5	0.87	0.23–3.33	0.84
Hypothermia at T6	1.41	0.48–4.20	0.53
Changes of temperature at T1	5.03	0.11–225.16	0.40
Changes of temperature at T2	8.54	0.69–104.96	0.09
Changes of temperature at T3	4.25	0.75–24.26	0.10
Changes of temperature at T4	7.60	0.84–68.73	0.07
Changes of temperature at T5	10.77	1.36–85.21	0.02*
Changes of temperature at T6	8.88	1.29–60.97	0.03*

^*^Was used to indicate the statistically significant difference (*P* value < 0.05)

*95% CI* 95% confidence interval, *SA* spinal-epidural anesthesia

**Fig. 2:**
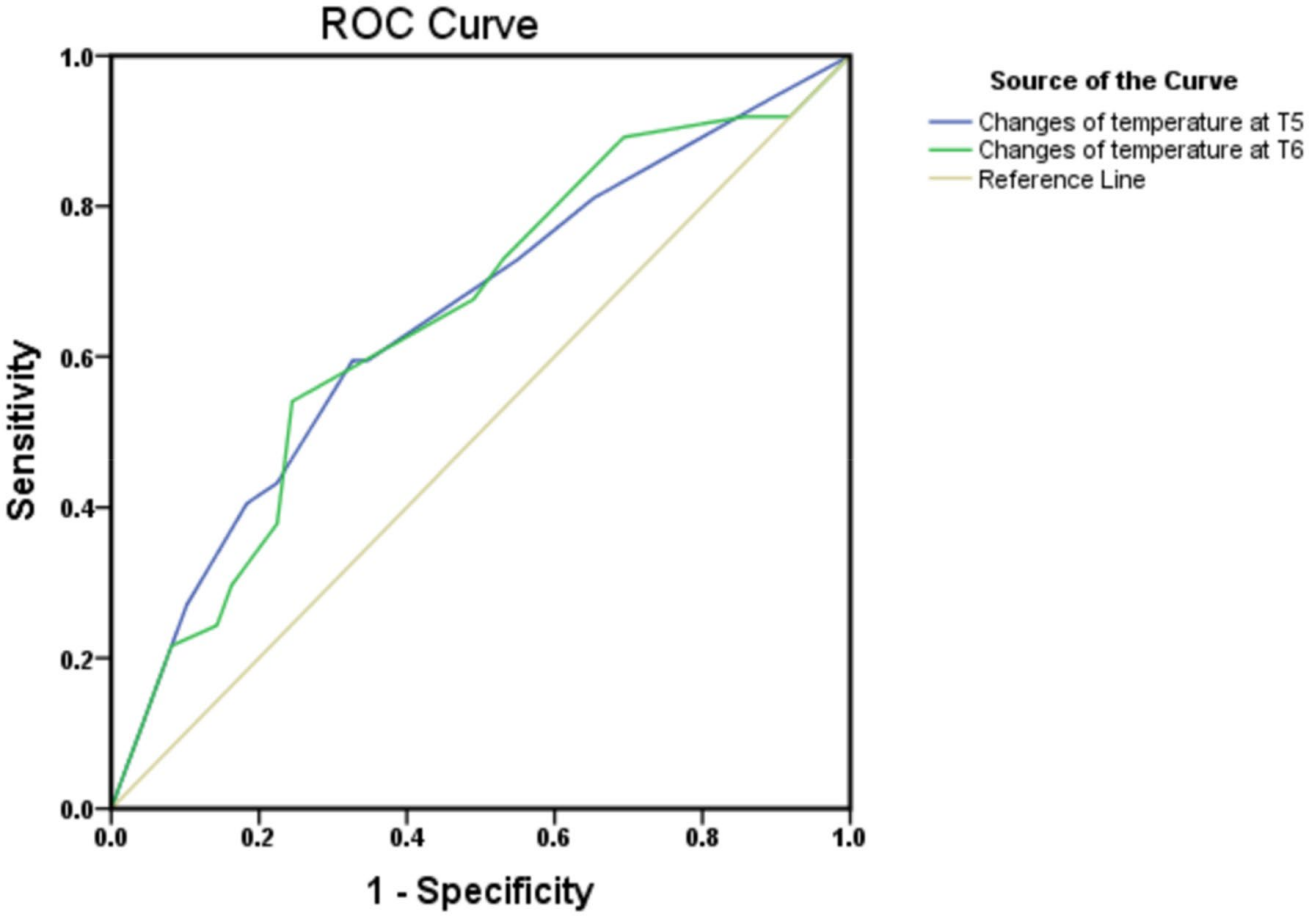
The ROC curve for prediction of shivering occurrence by core temperatures changes at T5 and T6

According to the temperature change of T5 and T6, the ROC curves were drawn respectively, and the critical point, sensitivity, specificity, and AUC were calculated for the temperature change to predict the occurrence of shivering (Table 5). The ROC curves with 1-specificity as the abscissa and sensitivity as the ordinate were shown in Fig. 2. It can be seen that the area under the ROC curve of changes of temperature at T5 and T6 was comparable.

**Table 5:** The critical point, sensitivity, specificity and AUC of core temperature change after SA

	Optimal critical point	Sensitivity	Specificity	AUC
Changes of temperature at T5	0.45	0.59	0.67	0.65
Changes of temperature at T6	0.55	0.54	0.76	0.65

*AUC* area under curve, *ROC* receiver operating characteristic curve

## Discussion

Shivering continues to be a common problem after spinal anesthesia for cesarean delivery, and we explored the association between shivering and hypothermia, temperature change, and local anesthetic.

In our study, changes of temperature at T5 and T6 were statistically associated with shivering, while hypothermia (core temperature < 36.0 ℃) after SA was not associated with shivering. Previous studies have demonstrated the mechanism of shivering caused by temperature decreases. When the core temperature decreases 0.5 ℃ or to the shivering threshold, peripheral thermosensors deliver the thermal information through the lateral spinothalamic tract to the preoptic area of the anterior hypothalamus, which is the thermoregulatory center. Then an area between the anterior and posterior hypothalamus, where the efferent shivering pathway starts, deliveries the reaction information to the reticular formation in the pons, leading to skeletal muscles involuntary and repetitive actions [17]. The ROC curve for prediction of shivering occurrence by core temperature 0.45–0.5 °C changes. Because shivering is a thermoregulatory reflex triggered by lower core temperature than the hypothalamic set point [18]. In addition, the lowest hypothermia was 35.1 °C in group B vs. 35.8 °C in group R. On the other hand, ropivacaine has less degree of motor blockade, less analgesic effect may be the other reason for less shivering. So the shivering occurrence was not significantly associated with the hypothermia (core temperature < 36.0 °C) in our study, but associated with the core temperature change or hypothalamic set point, which was over 0.45–0.5 °C. However, rectal temperature is less reliable because it lags behind core temperature during rapid thermal changes [19]. Nevertheless, the temperature in this study statistically decreased in only 10 min. In addition, the rectum is close to the surgical field of cesarean delivery. The measured temperature in this study could have been affected by surgical procedures. Therefore, we need more sensitive and accurate temperature monitoring in future research.

Neuraxial anesthesia, especially SA, is a suitable and common technique for caesarean delivery. The mechanism of high incidence shivering rate under spinal anesthesia may include heat loss with vasodilation below the level of sensory block, decrease of core-periphery temperature gradient, subsequent redistribution of blood, reduction of thermoregulatory vasoconstriction and shivering thresholds, and cold fluid infusion. Vasoconstriction above the dermatomal level of the sensory block does not appear to prevent decrease in core temperature [20–22]. The shivering incidence and intensity were high in group B; meanwhile, the severe shivering score only appears for bupivacaine. Our study also showed ropivacaine has a lower incidence of hypothermia than bupivacaine. Bupivacaine is an amino-amide local anesthetic introduced in 1963 and has been the standard local anesthetic used in obstetrics for many years. It relatively highly binds to α1-glycoprotein and has a long duration of action, both of which minimize the fetal dose. Its disadvantage is cardiac and central nervous system toxicity. The maximum safe dosage of bupivacaine is 2 mg•kg^−1^ [23]. Ropivacaine is the propyl homologue of bupivacaine (propivacaine) but is prepared as the pure S(–)-enantiomer [24]. Compared with bupivacaine, the shorter propyl (C3H7) substituent on the piperidine nitrogen atom of ropivacaine decreases lipid solubility and sodium channel affinity so does the duration of its action. Plentiful researches showed that 0.5% of bupivacaine was the same in sensory or motor blockade with 0.5 or 0.75% ropivacaine under epidural anesthesia. And bupivacaine rose and remained skin temperature 1 °C higher after five hours compared with basal values [25]. Isobaric 0.5% ropivacaine produces a similar duration of sensory block and a statistically significant shorter duration of motor block with 0.5% isobaric bupivacaine in lower abdominal surgeries [26]. Few studies have observed and analyzed the difference in the incidence of shivering caused by different local anesthetics during SA. We hypothesized that weaker motor block was associated with less risk of shivering because of the greater muscle control. Therefore, ropivacaine causes fewer shivering cases than bupivacaine in our study.

The MAP decreased in 5 min after intrathecal either bupivacaine or ropivacaine; it means the same sympathetic never block degree for 0.5% bupivacaine and 0.5% ropivacaine. After SA, ephedrine treatment for hypotension would interfere with the following measurement of blood pressure and heart rate. Nevertheless, only 3 cases were treated with ephedrine 5 ~ 10 mg because of hypotension (2 in group B and 1 in group R) in our study. The result showed that the dose of medication and maximum sensory block level was not statistically different among the two groups. If the difference of sensory nerve blockade levels between the two groups is significant due to different dosages, it may lead to misjudging the relationship between local anesthetic types and shivering. Though we have controlled the baricity, the temperature, concentration of each local anesthetic in our study, it should be cautious in explaining the shivering difference between the two groups.

Perioperative shivering could make misjudgment through interfering with monitoring of blood pressure, electrocardiogram, and pulse oximetry [3, 4]. It also increases plasma catecholamines and cardiac output; double oxygen consumption and carbon dioxide production [27, 28], and causes discomfort and dissatisfaction, especially during neuraxial anesthesia [7, 29]. There are multiple strategies to prevent intraoperative shivering. The researches show that appropriate use of dexmedetomidine, fentanyl, sufentanil, ketamine, meperidine, tramadol, and MgSO4 may effectively reduce the incidence and severity of shivering during cesarean section under neuraxial anesthesia [22], and body warming using a heated (maximum of 43 °C) forced-air blanket should be initiated [18]. Our study provides new evidence to guide clinical practice. In the process of body temperature monitoring, more attention should be paid to the change of body temperature rather than the specific value, and ropivacaine may be a better local anesthetics in the SA of cesarean section.

### Limitations

There are several important limitations of our study to highlight. First, this is a hypothesis study with only eighty-six parturients were included in the study, so the interpretation of our findings might be limited due to sample size. Further cohort studies or case–control studies based on a larger population are warranted to verify our results. Second, though we have controlled the baricity, the temperature, concentration of each local anesthetic in our study, the difference in sensory nerve blockade level between the two groups may interfere with the relationship between local anesthetic types and shivering. Third, because of our specific patients who were accepted caesarean section and specific anesthesia methods, our conclusions may not be generalizable to other populations.

## Conclusion

In our study, for caesarean section, the occurrence of shivering was associated with the local anesthetics, and the change of core temperature after SA, while not the hypothermia. Ropivacaine may lead to a lower incidence and intensity of shivering than bupivacaine. The greater change of core temperature from 25 to 30 min after SA, the more prone the parturient to shivering.

## Data Availability

The datasets used and/or analyzed during the current study are available from the corresponding author on reasonable request.

## Abbreviations

SA: Spinal-epidural anesthesia
ASA: American Society of Anaesthesiologist
MBP: Mean arterial pressure
SD: Standard deviation
